# Enhancing recombinant eGFP secretion in *P. tricornutum* by optimizing culture conditions

**DOI:** 10.1186/s12934-026-03061-3

**Published:** 2026-07-16

**Authors:** Nesrine Gargouch, Aurélie Charrier, Charlotte Toustou, Gaël Bougaran, Bruno Saint-Jean, Muriel Bardor

**Affiliations:** 1 Laboratoire GlycoMEV UR 4358, SFR Normandie Végétal FED 4277, Innovation Chimie Carnot, Université de Rouen Normandie, Rouen, 76000 France; 2 IFREMER, PHYTOX, Laboratoire PHYSALG, 44000 Nantes, France; 3 IFREMER, PHYTOX, Laboratoire GENALG, 44000 Nantes, France; 4 Alga Biologics, 25 Rue Tesnières, 76821 Mont Saint Aignan and 1 rue de l’industrie, 76380 Canteleu, France

**Keywords:** *Phaeodactylum tricornutum*, Green fluorescent protein, Culture medium, PPFD, pH

## Abstract

*Phaeodactylum tricornutum* is a promising candidate for the production of recombinant therapeutic proteins including antibodies. So far, low secretion yields remain a significant bottleneck for pharmaceutical applications. Towards the objective to increase production yield of secreted recombinant protein, this study investigates the optimization of environmental and nutritional parameters, under laboratory conditions, to maximize the production of a model secreted recombinant protein (eGFPsec) under the control of the Nitrate Reductase promoter (P_NR_). We evaluated the interplay between nitrate availability, Photosynthetic Photon Flux Density (PPFD), and pH level within both natural and artificial seawater matrices. Our results demonstrate that while P_NR_ activity does not increase with nitrate concentrations beyond non-limiting levels, a PPFD of 150 µmol photons m^− 2^ s^− 1^ significantly enhances eGFPsec accumulation in the medium. Interestingly, cultures grown in Natural Sea Water under pH-regulated conditions (pH = 8) exhibited markedly higher secreted-eGFP fluorescence compared to those in artificial seawater. These findings highlight the importance of optimizing key environmental and nutritional parameters to improve secretion recombinant protein yields. By identifying key drivers of eGFP fluorescence-based productivity and accumulation in the medium, this work provides actionable insights for process optimization, reinforcing the potential of *P. tricornutum* as a robust and cost-effective platform for industrial microalgae-based biomanufacturing.

**Supplementary Information:**

The online version contains supplementary material available at https://doi.org/10.1186/s12934-026-03061-3.

## Introduction

Over the past decades, microalgae have attracted increasing interest in various biotechnological processes, including food, feed, nutraceuticals, cosmetics, biofuel, and bioethanol, due to their ability to produce high-value molecules such as proteins, lipids, polysaccharides and pigments [1–4]. Recently, several scientific studies have shown that microalgae can also serve as efficient cell biofactories for the production of recombinant proteins [5], such as subunit vaccines [6], antimicrobial peptides [7], enzymes [8], and antibodies [9, 10]. In fact, they offer significant advantages over conventional eukaryotic expression systems, such as mammalian cells. Their growth rates are generally higher, and scale-up procedures are typically easier and less expensive [11, 12].

These promising production systems involve the use of diatoms which are eukaryotic and photosynthetic microorganisms that contribute to 20% of global oxygen production [13]. Among diatoms, *P. tricornutum* stands out as a well-characterized model [14]. It is a robust species on both laboratory and industrial scales [15] and can be grown in a wide range of inexpensive culture media [16]. The entire genomic sequence of *P. tricornutum* has been fully characterized [17, 18], and molecular tools, like plasmid delivery methods [19, 20], have been developed to enable genetic manipulation of this species. Moreover, *P. tricornutum* has the capability to produce complex recombinant proteins with correct folding and post-translational modifications, including *N-*glycosylation [21]. These modifications closely resemble the universal *N-*glycosylation pattern found in human proteins [22–24]. In addition, *P. tricornutum* also offers the advantage of enabling recombinant proteins to be secreted directly into the culture medium, which simplifies downstream processing (DSP) and reduces purification costs. These advancements have played a crucial role in the adoption of *P. tricornutum* as an alternative to conventional expression systems, such as mammalian cells, for recombinant protein production [25–27]. Recombinant protein production in *P. tricornutum* is generally conducted under controlled and confined cultivation conditions, particularly for pharmaceutical applications requiring high standards of safety, quality, and reproducibility. Moreover, selecting the appropriate promoter, a regulatory element that controls the initiation and efficiency of transcription, is one of the most critical steps in designing the expression vector for optimal transgene expression. To date, several promoters have been selected and used to produce recombinant proteins in *P. tricornutum* [28–34]. Among them, the inducible nitrate reductase promoter (P_NR_) stands out. This promoter is activated in the presence of nitrate ions and repressed in the presence of ammonium ions [35, 36]. Native nitrate-inducible promoters have been used in various algal species, such as *Cylindrotheca fusiformis* [36], *Nannochloropsis gaditana* [37], *Chlorella ellipsoidea* [38], *Dunaliella salina* [39], *Volvox carteri* [40], and *P. tricornutum* [41] for which it has been proven to efficiently drive the expression of foreign genes. For example, monoclonal antibodies against the hepatitis B virus surface protein [10, 42] and the nucleoprotein of the Marburg virus [43] have been successfully produced in the diatom *P. tricornutum* under the control of the inducible NR promoter. However, the production yield remains currently limited and uncompetitive with mammalian cell cultures for pharmaceutical applications [44]. Therefore, improving the production and secretion yields of recombinant proteins in *P. tricornutum* remains a challenge.

To date, most studies aimed at improving recombinant protein expression in microalgae have mainly focused on genetic engineering strategies, including promoter selection, codon optimization, and the integration of regulatory elements [44–47]. In contrast, the influence of cultivation conditions on recombinant protein expression remains comparatively less explored. Recently, optimization of environmental and nutritional parameters was shown to significantly enhance YFP production in *P. tricornutum* under the control of the HASP1 promoter [48]. However, studies investigating the impact of culture conditions on recombinant protein production and secretion driven by the inducible P_NR_ are still lacking.

In this context, the present study aims to evaluate the impact of key cultivation parameters, including nitrate availability, photosynthetic photon flux density (PPFD), pH, and culture medium composition, on the expression and accumulation in the medium of recombinant protein in *P. tricornutum* cultures under laboratory-scale conditions. eGFP was used as a reporter recombinant protein under the control of the P_NR_ and expressed as a secreted form (eGFPsec), allowing its accumulation in the culture medium. Fluorescence intensity and fluorescence-based productivity of secreted-eGFP (FBPS) were then monitored under varying culture conditions. By adopting a process-oriented approach, this study highlights the influence of environmental and nutritional parameters on recombinant protein accumulation in the medium, thereby supporting the development of *P. tricornutum* as an efficient platform for industrial microalgae-based bioproduction.

## Materials and methods

### Microalgae strain

*P. tricornutum* Pt1.8.6 (CAP 1055/1) later referring to Wild-Type (WT) was generously provided by the Physiology and Biotechnology of Algae (PBA) laboratory at IFREMER (Nantes, France) and clones expressing extracellular eGFP (eGFP_sec_) were obtained by transforming the WT strain through biolistic transformation as previously described [49]. Briefly, tungsten particles (1 μm) were coated with 5 µg of plasmid DNA using 50 µL of 2.5 M CaCl_2_ and 20 µL of 0.1 M spermidine, then accelerated toward diatom cells plated on agar using the particle Delivery System-1000/He (Bio-Rad) at a rupture pressure of 1350 psi under vacuum (25 Hg). After bombardment, cells were incubated for 48 h before being plated on selective agar medium containing zeocin (75 µg mL^− 1^). Resistant colonies were obtained after approximately 3–4 weeks, and were subculturated on fresh selective agar medium to isolate stable transformants.

Plasmids required for the genetic transformation of the microalga were designed, manufactured, and ordered from e-zyvec (Polyplus-Sartorius, France) (Figure S1). This vector contains a promoter and a terminator corresponding to the ones of the nitrate reductase (NR) gene, the zeocin resistance gene and the sequence encoding the fluorescent protein eGFP containing a signal peptide allowing the secretion and accumulation of the eGFP in the culture medium.

### Culture conditions

#### Effect of nitrate concentration

eGFP_sec_ strain was cultivated in synthetic seawater made from Salt Instant Ocean (33.3 g L^− 1^ of sea salt, Aquarium Systems, Sarrebourg, France) supplemented with 1 mL L^− 1^ of Conway nutritive medium, providing essential macronutriments, trace metals and vitamins [50]. Cultures were grown at two different concentrations of NaNO_3_ equal to 1.2 mM (Standard concentration [50]), and 12 mM.

The cultures were grown in 250 mL flasks containing 100 mL of culture medium, stirred at 150 rpm, at a temperature of 19 °C and under a photoperiod of 16 h of light followed by 8 h of darkness, with a PPFD of 30 µmol photons m^− 2^ s^− 1^. The flasks were incubated for 8 days in an Economic Lux Chamber (SNIJDERS LABS, Netherland). For each condition tested, cultures were performed in triplicate.

#### Effect of PPFD

eGFP_sec_ strain were cultivated in the Multi-cultivator MC 1000-OD-MIX (Photons System Instruments, PSI, Czech Republic) using synthetic seawater enriched with 1 mL L^− 1^ of standard Conway medium [50]. The cultures were conducted in 8 cultivation tubes, each filled with 80 mL of medium, and inoculated at an initial cell density of 6.10^6^ cells mL^− 1^ each, and were continuously bubbled with air at a constant flow rate, manually controlled *via* manifold valves. All the tubes were immersed in a temperature-controlled water bath at 19 °C. To test the impact of PPFD on the production of eGFP, 4 tubes were used. Each tube was exposed to continuous illumination at one of the four PPFD levels equal to 30, 90, 150 and 250 µmol photons m^− 2^ s^− 1^.

#### Effect of natural and synthetic seawater media and pH regulation

eGFP_sec_ strain was acclimated for 7 days in 200 ml flasks of natural or synthetic seawater enriched with Conway medium [50] with ammonium replacing nitrate as source of nitrogen. All cultures were maintained at a constant temperature of 20 °C, under a constant PPFD of 150 µmol photons m^− 2^ s^− 1^. After acclimation, strains were phenotyped using a phenotyping bench [51] for 7 days. For this, eGFP_sec_ strains was inoculated at a density of 1.10^6^ cells mL^− 1^ in photobioreactors containing 200 ml of either natural or synthetic seawater, supplemented with Conway medium enriched one- or two-fold and with nitrate as source of nitrogen required to induce the expression of the recombinant eGFP protein in the eGFP_sec_ strain. The concentration of Conway medium - whether single or double- had no effect on growth rate of recombinant strains or on eGFP protein expression (data not shown). Culture conditions were kept constant and identical among PBRs with 0.22 μm filtered air bubbling, PPFD (150 µmol photons m^− 2^ s^− 1^), temperature (20 °C) and unregulated or regulated pH (at pH 8) with CO_2_ injection. Cultures were sampled every day along the 7 days cultures for cell density and eGFP excretion measurements. For each condition tested, cultures were performed in triplicate.

### Cellular and biochemical analyses carried out on microalgae cultures under different nitrate concentration and PPFD

#### Cell density and nitrate measurement

Microalgae growth was monitored by cell counting using a Countess II Automated Cell Counter (Thermo Fisher). Nitrate consumption was determined using a modified colorimetric method [52]. Briefly, culture supernatants obtained after centrifugation (5,000 × g, 5 min) were treated with perchloric acid, and absorbance was measured at 210 and 275 nm to correct for potential interferences. Nitrate concentration was calculated using a corrected absorbance equation.

$$ \begin{gathered} {\mathrm{A}}_{{{\mathrm{corrected}}}} \hfill \\ = {\text{ A}}_{{{\mathrm{21}}0}} - {\text{ 2 x }}({\mathrm{A}}_{{{\mathrm{275}}}} - {\text{ Amoy}}_{{{\mathrm{275}}/{\text{Standard range}}}} ) \hfill \\ \end{gathered} $$

with : 

A_210_: sample absorbance at 210 nm

A_275_: sample absorbance at 275 nm

Amoy_275/Standard range_: average absorbance of the standard range at 275 nm

#### Fluorescence measurement of eGFP in cell-free medium

Fluorescence measurements performed directly on the total culture were excluded from quantitative analyses because cell shading significantly affected the detected eGFP signal. The extent of fluorescence attenuation varied with biomass concentration, leading to inconsistent and non-quantifiable measurements. To avoid this bias, only eGFP fluorescence measured in the extracellular medium (cell free medium) was used for data analysis and interpretation. 150 µL of supernatant obtained after culture centrifugation at 5,000 x g for 5 min (cell free medium), were loaded in triplicate into a black clear-bottom 96-well plate (Costar). The measurement of eGFP fluorescence was performed by exciting the protein at a wavelength of 485 (20) nm, and the emission was recorded at 538 (20) nm using a fluorometer (FlexStation 3 MultiMode Microplate Reader, Molecular devices). The auto-fluorescence of the WT was subtracted from each condition to remove the chlorophyll background signal and FBPS was calculated daily for the transformed strain according to the following equation:

$$ {\text{FBPS }}({\mathrm{a}}.{\mathrm{u}}.{\text{ day}}^{{ - \,{\mathrm{1}}}} ){\text{ }} = {\text{ }}{{{\text{Fluorescence intensity}}} \mathord{\left/ {\vphantom {{{\text{Fluorescence intensity}}} {{\text{Elapsed time}}}}} \right. \kern-\nulldelimiterspace} {{\text{Elapsed time}}}} $$

#### Protein extraction

To recover eGFPsec accumulated in the culture medium, extracellular proteins were extracted from the supernatant obtained after centrifugation of 2 mL of microalgal culture at 5,000 x g for 5 min. Total secreted proteins were precipitated from the recovered culture supernatant using a mixture of TCA and NP40, followed by centrifugation to collect protein pellets. The pellets were washed with cold acetone and air-dried prior to further analysis.

#### Western blot analysis of recombinant protein expression

The presence of eGFP in the supernatant was analyzed by Western blot under reducing conditions. Briefly, protein extracts were separated on a 12% Tris- glycine SDS-PAGE and transferred to a nitrocellulose membrane. After saturation with TBS-T buffer containing 5% skim milk, the membranes were then incubated with a sheep anti-GFP polyclonal antibody (Invitrogen) diluted at 1: 2000 in TBS-T buffer with 1% skim milk for 2 h at room temperature. After washing, the membranes were incubated with Horseradish peroxidase (HRP)-conjugated donkey anti-sheep antibody (Invitrogen) diluted at 1: 20,000 in TBS-T buffer for 1 h at room temperature. Finally, immunoblot signals were detected using the ECL Pico Plus kit (SuperSignalTM West Pico PLUS Chemiluminescent Substrate, Thermofisher) following the manufacturer’s instructions. Purified eGFP, produced in *Arabidopsis thaliana* root hairs, was used as a positive control [53], and protein extract from the WT strain was used as a negative control.

### Cellular and biochemical analyses carried out on microalgae cultures under different seawater media and pH control

#### Cell density measurement

Cell concentration was assessed using Coulter Counter Multisizer 4 (Beckman Coulter, High Wycombe, UK). Before measurement, samples were diluted to 1/50 or 1/100 with sterile seawater to allow maximum detection of the number of cells. The size of the cells counted was between 3 and 7 μm. Cell number, given as equivalent sphere diameter, was then calculated using MS-Multisizer 4 software (Beckman Coulter, High Wycombe, UK).

#### eGFP fluorescence measurements in cell-free medium

Fluorescence measurements performed directly on the total culture were excluded from quantitative analyses because cell shading significantly affected the detected eGFP signal. The extent of fluorescence attenuation varied with biomass concentration, leading to inconsistent and non-quantifiable measurements. To avoid this bias, only eGFP fluorescence measured in the extracellular medium (cell free medium) was used for data analysis and interpretation.

To evaluate eGFP expression from extracellular medium, the supernatant (cell free medium) was obtained by centrifugation of 1 mL of culture at 1,500 x g for 15 min in 1.5 mL Eppendorff tube. 100 µL of supernatant were transferred in triplicate onto a black NUNC 96-well clear-bottom microplate (NUNC, Thermo Fisher Scientific). In order to control there was no cell lysis contamination, chlorophyll *a* fluorescence was measured in the cell free medium using 450 (9) nm excitation and 685 (20) nm emission respectively.

### Statistical analysis

Statistical analyses were performed in RStudio.

## Results and discussion

### Impact of nitrate concentration

The P_NR_ is particularly effective for inducible expression of recombinant proteins, especially during the transition from an ammonium to a nitrate containing medium [10, 36, 41]. Therefore, we investigated the response of the P_NR_ to different NO_3_^−^ ion concentrations and their effects on the production yield of the secreted recombinant protein eGFPsec accumulated in the culture medium. To this end, the eGFP_sec_ strain was cultured in Instant Ocean seawater-based medium supplemented with Conway’s solution containing two concentrations of NaNO_3_, 1.2 and 12 mM, respectively. The induction of the P_NR_ is commonly achieved through a switch from NH_4_^+^ to NO_3_^−^, which can be performed either at the beginning or the end of growth phase in batch cultures to trigger eGFP production. However, when NH_4_^+^ is used as the primary nitrogen source for full culture growth, the subsequent increase in pH can lead to ammonia (NH_3_) production, which is highly toxic to microalgae [54]. This necessitates strict pH control. Thus, in this study, we opted for an early switch to NO_3_^−^ to promote the continuous accumulation of eGFP in the medium, thereby maximizing total yield.

**Fig. 1:**
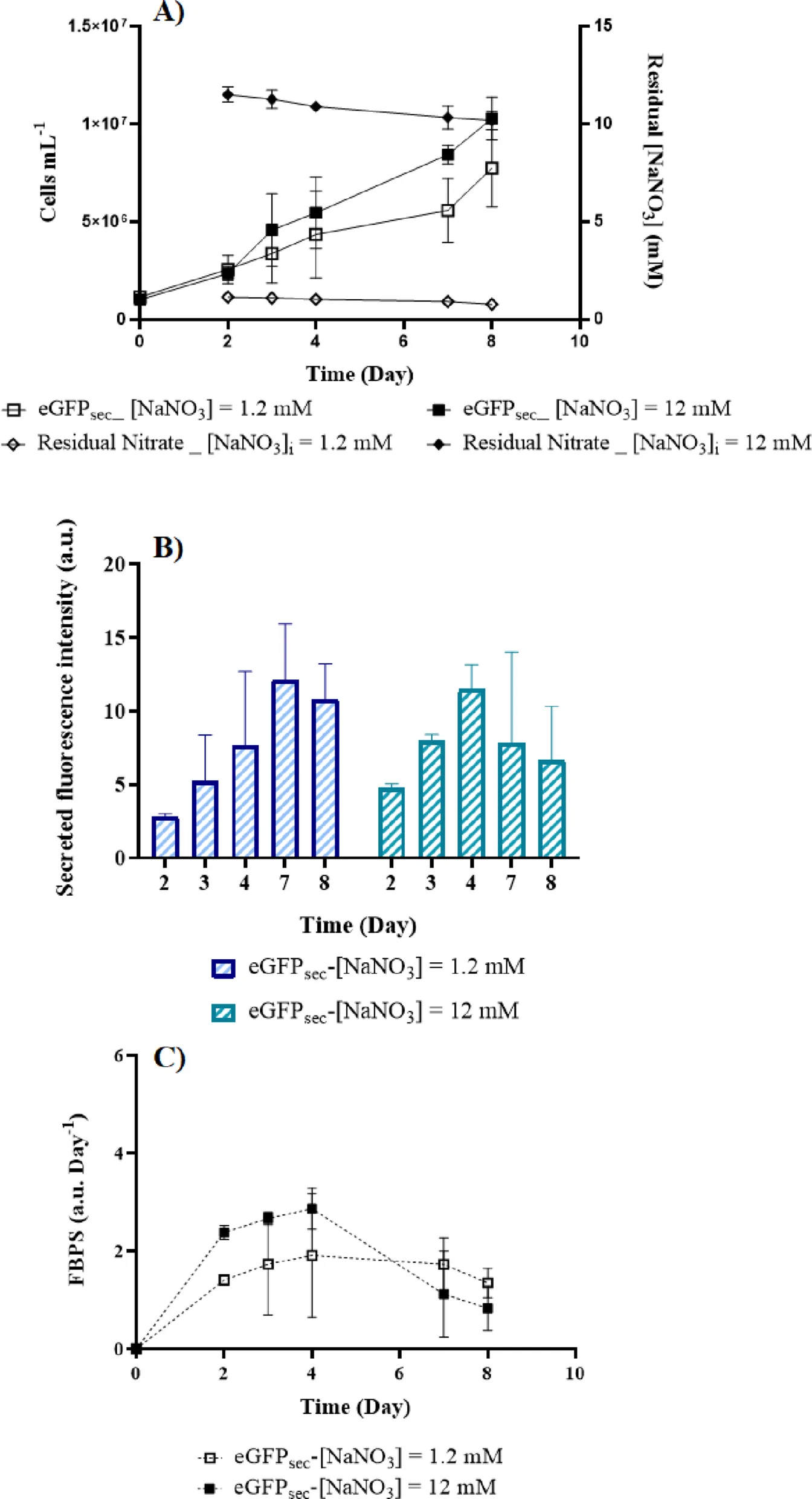
Effect of nitrate concentration on growth, eGFPsec accumulation in the medium, and fluorescence-based productivity of secreted eGFP (FBPS) of the eGFP_sec_ strain cultivated in Instant Ocean medium. **A** Growth kinetics of eGFP_sec_ strain cultivated in Salt Instant Ocean media supplemented with Conway medium containing either 1.2 mM « □ » or 12 mM « ■ » NaNO_3_. Residual nitrate concentrations in the culture medium are represented by « ◇ » for 1.2 Mm NaNO3 and « ♦ » for 12 Mm NaNO_3_. **B** secreted eGFP fluorescence intensity measured in cell-free medium of cultures supplemented with 1.2 mM (blue bar histogram) or 12 mM (teal bar histogram) NaNO_3_. **C** FBPS measured in the cell-free medium

Over a period of 8 days, we monitored the growth kinetics (Fig. 1A) and computed the specific growth rates (µ_max_) for eGFP_sec_ strain under the two nitrate concentrations. Increasing nitrate availability was associated with a slight increase in growth rate, from 0.35 ± 0.12 d⁻^1^ to 0.47 ± 0.03 d⁻^1^. However, ANOVA analysis (Table S1) did not reveal a statistically significant effect of nitrate enrichment on the growth rate of the eGFP_sec_ strain under the tested conditions (*P* = 0.309), despite the apparent increase in mean growth rate.

The residual nitrate concentration in the culture medium of the eGFP_sec_ strain was also monitored over time (Fig. 1A). Our findings indicated that under the specified growth conditions used (light intensity of approximately 30 µmol photons m^− 2^ s^− 1^, temperature of 19 °C, and salinity of 33.3 g L^− 1^), NaNO_3_ was not a limiting factor. At day 8, the residual concentrations were 0.8 mM and 10 mM, respectively when the initial concentrations of NaNO_3_ in the culture medium were 1.2 mM and 12 mM. This finding suggests that the experimental conditions provided sufficient nitrate to support the activation of the NR promoter. As a result, these conditions help prevent the synthesis of undesirable or competitive metabolites, such as lipids [55], as well as unexpected metabolic responses associated with nitrogen limitation or deficiency. It is worth noting that under the high NO_3_^−^ enrichment, cells consumed 5 times more NO_3_^−^ than under the low condition. This resulted in a 3 times higher yield of NO_3_^−^ to build new cells when fed 1.2 mM NO_3_^−^. This discrepancy can be accounted for by the ability of cells to store nitrogen under large excess of external NO_3_^−^, but also by excretion of organic nitrogen by cells depending on their nutritional status, particularly when the supply of mineral nitrogen is highly excessive [56].

The Curves were plotted using GraphPad Prism software (version 8.0.2) and error bars indicate standard deviation calculated from three independent biological replicates (*n* = 3).

Then, we measured fluorescence intensity (Fig. 1B) and FBPS (Fig. 1C) in cell-free medium. No difference was observed between the two NaNO_3_ concentrations in either volumetric or cell-normalized eGFP fluorescence (Figure S1) measured as cumulative extracellular eGFP recovered in the cell-free medium. These results suggest that increasing nitrate availability beyond 1.2 mM did not further enhance recombinant protein expression and accumulation in extracellular medium under the tested conditions and may reflect saturation of the regulatory system controlling P_NR_ activity. Since eGFP fluorescence was used in the present study as a reporter of protein expression, it is to note that these observations provide indirect evidence of P_NR_ regulation.

Previous studies have shown that in plants, fungi, and microalgae, the nitrate reductase genes are expressed in response to very low levels of intracellular nitrate accumulation [40, 57], which depends directly on transport activity. For example, study on *Chlamydomonas reinhardtii* has shown that the saturation of the nitrate transporter systems has been associated with a plateau in signaling and stabilization of the activity of the nitrate reductase gene (*Nia1*) promoter [58]. In higher plants and microalgae, NO₃⁻ ions uptake are mediated by distinct systems: the high-affinity transport system (HATS) and the low-affinity transport system (LATS) [58, 59]. Previous studies have shown that the activity of these transport systems is regulated by nitrate availability and intracellular nitrogen status, resulting in feedback modulation of nitrate uptake and assimilation [60–62]. In the present study, the stabilization of eGFP fluorescence observed at 1.2 mM NaNO_3_ and the absence of differences in fluorescence intensity between the two nitrate concentrations, may thus be consistent with the high sensitivity of nitrate-responsive regulatory systems. In this context, future investigation of nitrate uptake dynamics, signaling pathways and transcriptional responses could provide further insight into their contribution to P_NR_ regulation and recombinant protein production in *P. tricornutum*. From a process perspective, since nitrate is required not only for promoter induction but also to sustain biomass formation, maintaining nitrate concentrations within an optimal range or exploring controlled nitrate-feeding strategies, such as fed-batch nitrate supply, may represent potential approaches to better balance cell growth and inducible recombinant protein expression.

As shown in Fig. 1C, FBPS measured in the cell-free medium increased up to day four, and then appeared to remain steady or slightly decrease from day 7 onward, while cell number still increased (Fig. 1A). This FBPS trend may reflect either limited accumulation efficiency in *P. tricornutum* during the last days of culture or degradation of the recombinant protein in the extracellular medium. Although proteolytic activity was not directly assessed in this study, previous reports have demonstrated the presence of extracellular proteases in *P. tricornutum* that can affect recombinant protein stability [63]. In addition, inhibition of proteolytic activity has been shown to improve the stability of recombinant proteins in *P. tricornutum* [64]. These observations suggest that extracellular proteolytic activity may contribute to reduce recombinant protein accumulation and highlight the importance of maintaining recombinant protein stability in the culture medium during process optimization. While protease inhibitors can improve protein stability, their use may be costly and impractical at large scale. Therefore, more sustainable strategies should be considered, including the identification of key extracellular proteases and their targeted knockdown or knockout using genetic approaches. Recent advances in CRISPR/Cas9 genome editing in diatoms [65] provide promising tools to engineer strains with reduced proteolytic activity.

### Effects of PPFD

Several factors significantly influence cellular growth and metabolic pathways of microalgae. Among these, light is particularly important as it serves as the primary energy source for photosynthesis and directly affects both the growth of microalgae and their biochemical composition [66, 67]. In this context, we cultured eGFP_sec_ strain in a multicultivator under different PPFD: 30, 90, 150, and 250 µmol photons m^− 2^ s^− 1^ and under continuous light. The multicultivator device provides better control of PPFD compared to the Economic Lux Chamber, ensuring uniform exposure across culture tubes. For these experiments and based on our previous results, we used the synthetic seawater enriched with standard Conway medium at a NaNO_3_ concentration of 1.2 mM.

**Fig. 2:**
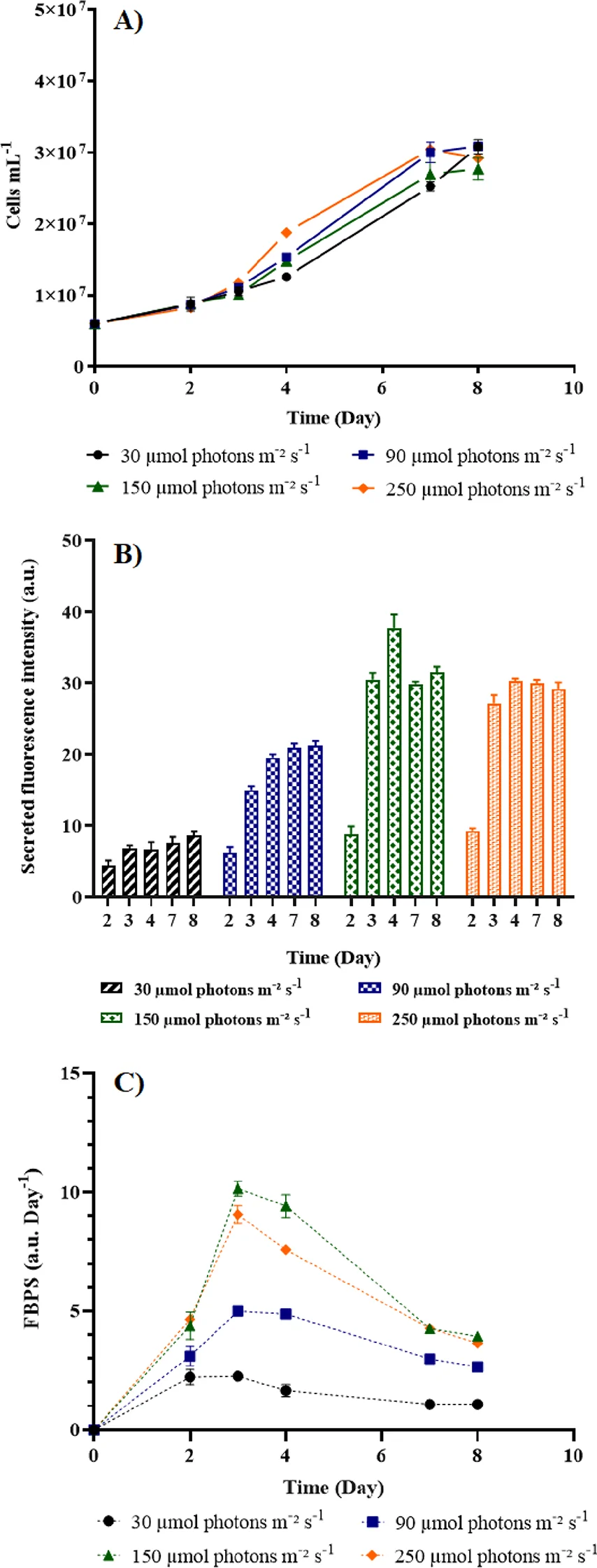
Effect of PPFD intensity on growth, eGFPsec accumulation in the medium, and secreted fluorescence-based productivity (FBPS) of the eGFP_sec_ strain cultivated in Instant Ocean medium. **A** Growth kinetics of eGFP_sec_ strain cultivated in Salt Instant Ocean media supplemented with Conway medium containing either 1.2 mM NaNO_3_ under different PPFD 30, 90, 150 and 250 µmol photons m^− 2^ s^− 1^. **B** eGFP fluorescence intensity measured in cell-free medium. **C** FBPS measured in cell-free medium

Cell density was monitored for 8 days (Fig. 2A) and µ_max_ was determined under the different PPFD (Table 1).

**Table 1:** Growth rates (µ_max_) of eGFP_sec_ strain at different PPFD

Irradiance (µmol photons m^− 2^ s^− 1^)	µ_max_ (d^− 1^)
30	0.18
90	0.29
150	0.26
250	0.41

Our results showed that the growth rate increased with PPFD, with µ_max_ reaching its highest value at 250 µmol photons m^− 2^ s^− 1^ (Table 1), whereas final cell concentrations remained relatively similar across different PPFD conditions (Fig. 2A). Similar responses have been reported in other microalgae, where increasing PPFD enhances photosynthetic activity and specific growth rates, while final biomass accumulation remains dependent on the interaction of multiple environmental and nutritional factors, including nutrient availability and self-shading [67–69]. Beyond its effect on growth kinetics, the impact of PPFD on eGFP accumulation in the medium was then evaluated.

Growth curves were plotted using GraphPad Prism software (version 8.0.2) and error bars indicate standard deviation calculated from three independent technical replicates (*n* = 3).

**Fig. 3:**
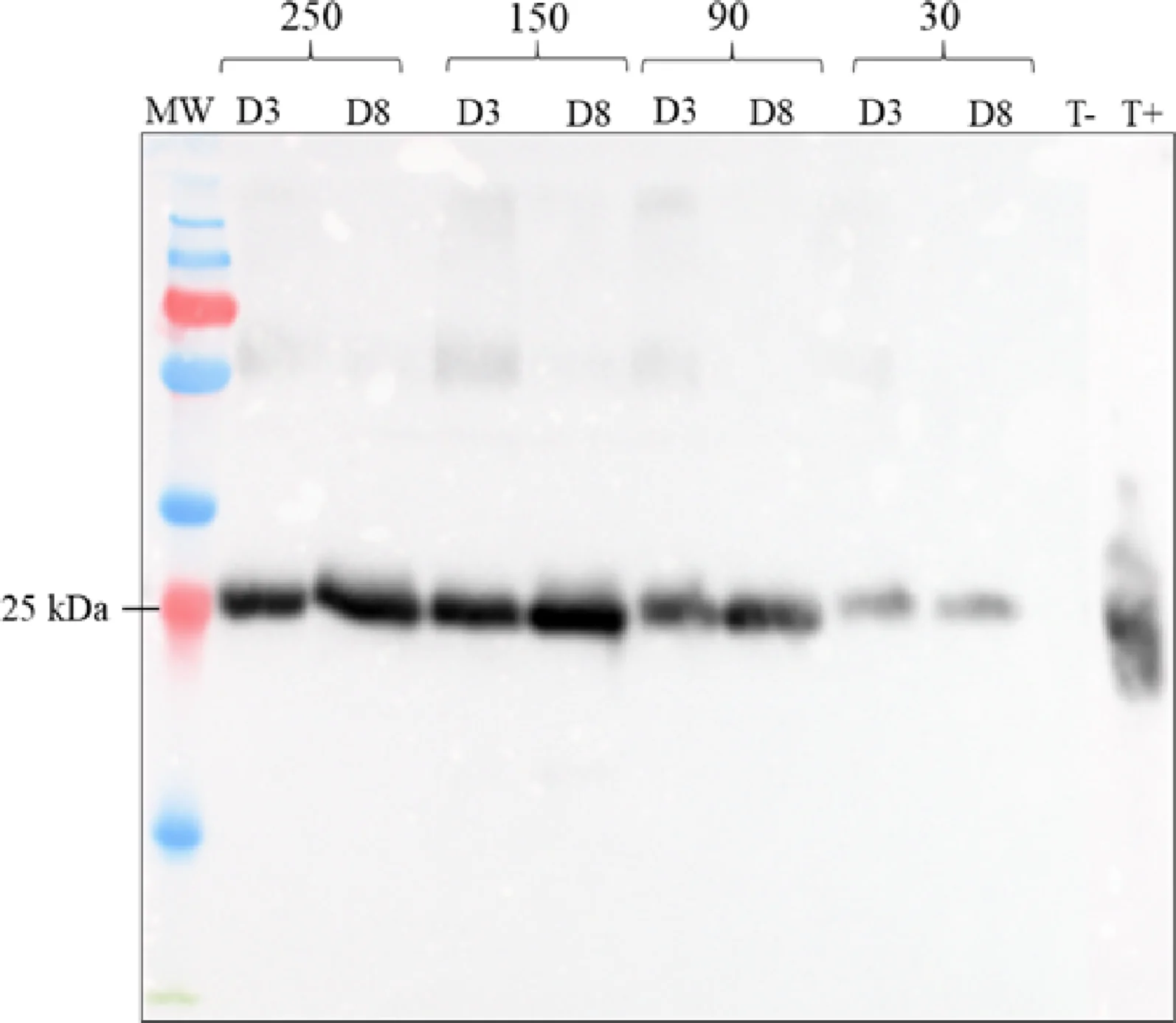
Immunodetection of extracellular eGFP in cell-free culture medium under different PPFD (250, 150, 90 and 30 µmol photons m^− 2^ s^− 1^). Western blot analysis performed using an anti-eGFP antibody on samples collected at days 3 (D3) and 8 (D8). Extracellular protein fractions recovered from the corresponding cell-free medium after TCA precipitation were prepared from the same initial culture volume (2 mL) and the entire recovered fractions were loaded onto the gel. MW: Molecular Weight marker, T-: Wild-type used as a negative control, T+: eGFP used as a positive control

Our results showed no photobleaching effect in either volumetric (Fig. 2B) or cell-normalized eGFP fluorescence (Figure S2) under high PPFD conditions. Instead, extracellular eGFP accumulation as measured by fluorescence in the cell-free medium, increased markedly with PPFD and reached its highest values under 150 µmol photons m⁻² s⁻¹ (Fig. 2B). Consistent with these observations, FBPS reached its maximum on day 3, with 10 a.u. day^− 1^ in the cell-free medium (Fig. 2C). These results indicate that increased PPFD promoted the accumulation of eGFP in the culture medium. Western blot analyses performed using an anti-eGFP antibody provided qualitative support for eGFP accumulation in the medium, with immunodetectable eGFP observed in extracellular fractions recovered from the culture medium under all PPFD conditions (Fig. 3). Overall, the Western blot profiles qualitatively agreed with accumulation of eGFP fluorescence measurements in the medium.

Few studies have investigated the effect of light intensity on the production of recombinant proteins in microalgae. However, it was already demonstrated that the results obtained for the same promoter and the expression of the same recombinant protein can vary considerably depending on the culture conditions. Erdene-Ochir et al. [32] demonstrated that the relative performance of CIP1 and fcpA promoters depended on light conditions, with CIP1-driven expression increasing under higher PPFD. In contrast, a recent multifactorial screening investigating factors influencing recombinant YFP production in *P. tricornutum* under the control of the HASP1 promoter did not identify PPFD as a statistically significant factor affecting fluorescence production [48]. They found that in combination with temperature, nitrate and NaCl concentrations, an optimal PPFD of 78.2 µmol photons m^− 2^ s^− 1^ was predicted for YFP production, slightly higher than standard culture conditions (75 µmol photons m^− 2^ s^− 1^).

Together with the present study, these findings suggest that environmental and nutritional conditions for the production of recombinant proteins must be optimized on a case-by-case basis for each promoter, strain and associated target protein. Our observations highlight the importance of optimizing especially the interaction between PPFD and nutrient concentration, for the expression, production and secretion of recombinant proteins in *P. tricornutum* under the control of P_NR_.

### Assessment of two types of seawater and pH regulation

The large-scale production of microalgae requires substantial resource inputs, with water being a key component. Notably, Natural Sea Water presents a promising source as a reference growth medium due to its richness in essential macro- and micronutrients, which promote optimal microalgal growth. In this context, we then assess the impact of Natural Sea Water on the accumulation of recombinant eGFP_sec_ in the medium in *P. tricornutum*. eGFP_sec_ strain was cultivated in Natural Sea Water collected from Saint-Malo, France, as compared to a culture of the same strain in reconstituted Instant Ocean salt with water. All cultivation processes were conducted as described in Sect.  2.2.3. As natural seawater can contain inorganic nitrogen, their concentrations have been routinely monitored at the natural seawater sampling site over the past six years. Mean ammonium and nitrate concentrations were 6.49 ± 1.51 µmol L⁻¹ and 48.86 ± 9.80 µmol L⁻¹, respectively, with limited temporal variability throughout the monitoring period. These background nitrogen concentrations are substantially lower than those provided through the culture medium (Conway) and therefore represent only a negligible consistent source of nitrogen. Consequently, they are unlikely to have influenced microalgal growth or affected the interpretation of the experimental results.

The use of Natural Sea Water for the cultivation of marine microalgae can lead to precipitation issues at high pH levels, which can hinder the growth of microalgae by forming undesirable deposits that disrupt nutrient absorption [70]. pH regulation through controlled CO₂ injection provides better control of cultivation conditions while maintaining the availability of inorganic carbon for photosynthesis and nutrient assimilation [71]. Maintaining a stable pH is also essential, as it supports optimal enzymatic activity and enhances metabolic efficiency in microalgae [72]. In this context, we used automated CO_2_ injection to maintain pH at a constant value of 8 during cultivation.

**Fig. 4:**
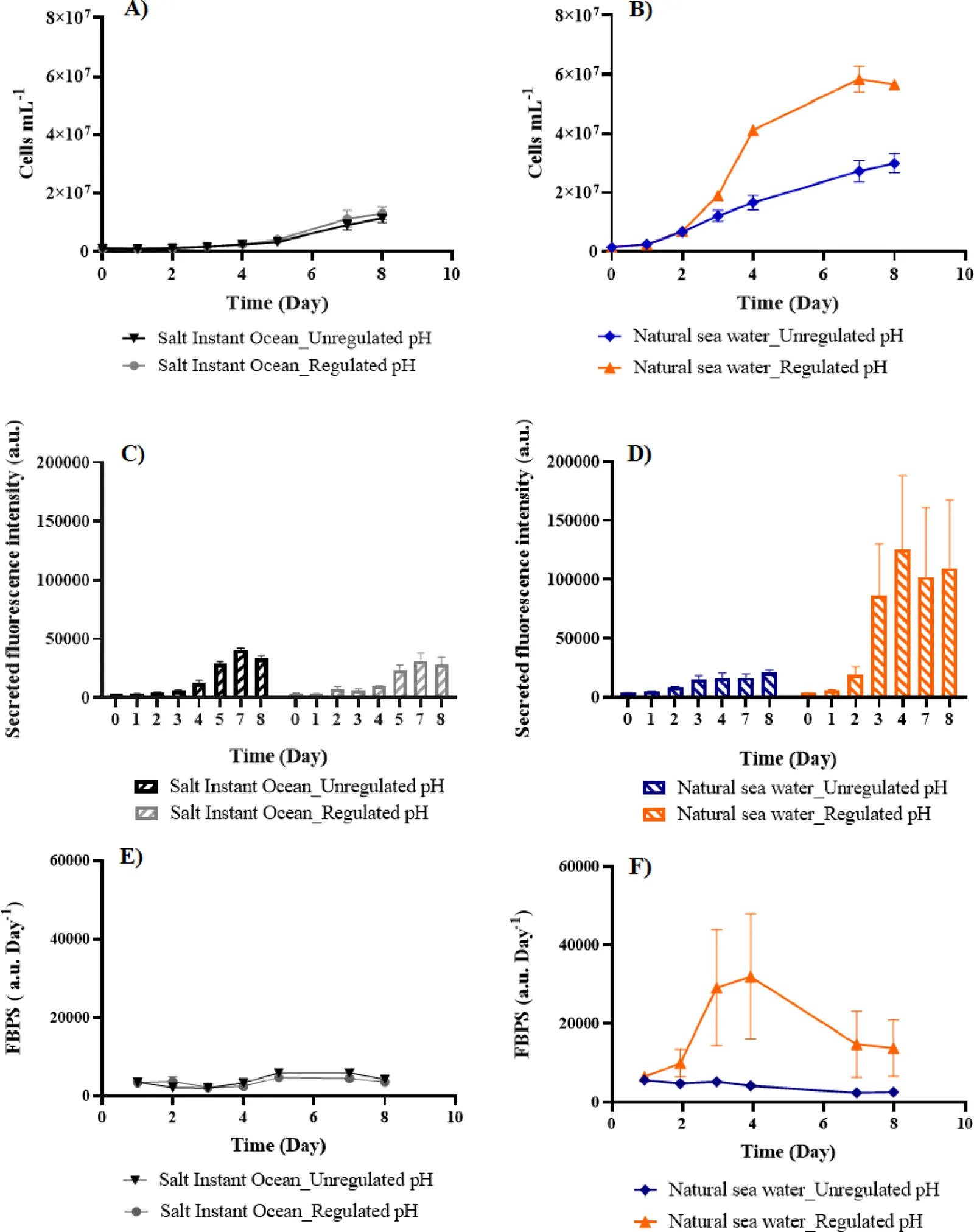
Growth of the eGFPsec strain under different culture media and pH conditions and their effects on eGFP accumulation in the medium culture. Growth kinetics of the eGFPsec strain cultivated in Instant Ocean medium (**A**) and Natural Sea Water (**B**) under unregulated and pH-regulated conditions (pH = 8.0). Extracellular eGFP fluorescence measured in cell-free culture medium from cultures grown in Instant Ocean (**C**) and Natural Sea Water (**D**) under unregulated and pH-regulated conditions. Secreted fluorescence-based productivity (FBPS), calculated from extracellular eGFP fluorescence measured in the cell-free medium, in cultures grown in Instant Ocean (**E**) and Natural Sea Water **(F)** under unregulated and pH-regulated conditions

As shown in Fig. 4A and B, the growth kinetics of the eGFP_sec_ strain cultivated in Natural Sea Water and Instant Ocean medium with and without pH regulation show clear differences in growth behavior. Natural seawater clearly enhanced microalgal growth, as evidenced by higher cell densities. The eGFP_sec_ strain reaches cell densities of 6.10^7^ cells mL^− 1^ with pH regulation and 3.10^7^ cells mL^− 1^ without pH regulation (final pH ranging from 10.1 to 10.3) in Natural Sea Water (Fig. 4B). In contrast, cultures grown in Instant Ocean medium, whether pH-regulated or not regulated (final pH ranging from 8.4 to 8.6), reached only 1.10^7^ cells mL^− 1^ (Fig. 4A). Our findings are in agreement with those of Lelekov et al. [73], who reported enhanced growth of *P. tricornutum* in Trenkenshu medium prepared with Black-sea seawater compared with artificial seawater. The authors attributed this difference to the availability of biogenic elements present in Natural Sea Water but limited or absent in artificial formulations. Furthermore, the interaction effect on *µ*_*max*_ of water type and pH regulation mode was highly significant (*p* = 0.00016). While pH regulation in Natural Sea Water increased *µ*_*max*_ by a factor of 1.48, the effect was much lower in Instant Ocean medium, being 1.1 only. This result demonstrated that the effect of pH regulation highly depends on the type of water used. Moreover, the specific growth rates were higher in Natural Sea Water in both pH-regulated and pH-unregulated cultures (*p* < 0.0001 and *p* = 0.0002, respectively), resulting in *µ*_*max*_ of 1.23 ± 0.01 and 0.83 ± 0.07 d^− 1^, respectively. In contrast, Instant Ocean medium supported lower growth rates, with µ_max_ values of 0.54 ± 0.05 d⁻¹ (with pH regulation) and 0.49 ± 0.06 d⁻¹ (without pH regulation). It is worth noting that in the light experiment the culture grown on Instant Ocean reached a higher final cell concentration (approximately 3.10⁷ cells mL⁻¹, compared with only 1.10⁷ cells mL⁻¹ in the present experiment). However, the difference in initial inoculum density (6.10⁶ and 1.10⁶ cells mL⁻¹, respectively), together with differences in the experimental protocols (e.g., light path length, mixing intensity, and PPFD), makes direct comparison between the two experiments challenging. In the end, these observations demonstrate that the growth conditions of the transformed strain are significantly improved by the use of Natural Sea Water in combination with the maintenance of a stable pH, equal to 8, throughout the culture.

Natural Sea Water not only promoted the cell growth of *P. tricornutum* but also resulted in a fivefold increased eGFP fluorescence intensity compared to artificial seawater (Salt Instant Ocean), as shown in Figs. 4C and D. This enhancement was especially pronounced when the pH was maintained at 8, highlighting the importance of both Sea water composition and pH regulation in optimizing recombinant protein accumulation in the medium culture. In contrast, pH regulation had no significant effect on eGFP accumulation in the medium in cultures grown in Instant Ocean (Fig. 4C). Fluorescence values in cell-free medium were additionally normalized to cell density (Figure S3). Interestingly, although Natural Sea Water supported greater volumetric eGFP fluorescence, higher cell-normalized fluorescence intensities were observed in Instant Ocean cultures. Thus, Natural Sea Water favored culture-scale productivity and extracellular eGFP recovery, whereas Instant Ocean cultures exhibited relatively higher eGFP fluorescence on a per-cell basis despite lower cell growth.

Growth curves were plotted using GraphPad Prism software (version 8.0.2) and error bars indicate standard deviation calculated from two independent technical replicates (*n* = 3).

Furthermore, cultures grown in Natural Sea Water exhibited higher FBPS than those grown in Instant Ocean medium, particularly under pH-regulated conditions (Fig. 4E, F). This increase in volumetric productivity of secreted eGFP is consistent with enhanced biomass accumulation under these conditions. In contrast, cultures grown in Instant Ocean showed relatively low and stable FBPS over time, with no difference observed between pH-regulated and unregulated conditions (Fig. 4E).

In this study, we evaluated the effects of natural and artificial (Salt Instant Ocean) sea water on the growth of *P. tricornutum* and the production of a secreted recombinant eGFP protein. Our findings show that, from a culture-scale perspective, Natural Sea Water, especially under pH-regulated conditions, promotes cell growth and enhances extracellular eGFP fluorescence compared to artificial seawater. The underlying factors for this enhanced performance are likely multifaceted. While the presence of a broader spectrum of trace elements and micronutrients in Natural Sea Water may support optimal cellular activity, its intrinsic complexity makes direct causal attribution challenging. Unlike the defined composition of artificial sea water, Natural Sea Water contains a diverse pool of dissolved organic matter (DOM) and natural ligands that can significantly influence nutrient speciation and bioavailability. These synergistic effects may facilitate not only biomass accumulation but also the metabolic pathways involved in protein production and/or secretion. Beyond biological performance, Natural Sea Water offers clear practical and economic advantages by reducing production costs for large-scale applications. For these reasons, it is an attractive option for sustainable microalgae bioprocesses. However, artificial sea water such as Salt Instant Ocean plays an equally important role in industrial settings. Its defined and reproducible composition allows precise control of culture conditions, ensuring experimental consistency and process standardization. This is especially important in biotechnology workflows where reproducibility and GMP regulatory compliance are key. In fact, Natural Sea Water can be seen as the ideal reference for microalgal growth, as it provides a natural balance of nutrients that supports high cell performance. So, it can be used as a goal to guide the improvement of artificial seawater composition. By identifying which components in Natural Sea Water are responsible for better growth and accumulation in the medium, we can adapt artificial media to be more effective. This way, it becomes possible to combine the high productivity of Natural Sea Water with the experimental control and reliability of artificial media.

## Conclusion

In this study, we evaluated for the first time the impact of culture conditions on P_NR_ -driven recombinant protein expression in *P. tricornutum* and accumulation in the medium. Although P_NR_ is active when nitrogen is present as nitrate ions, our results showed that a low (NaNO_3_ = 1.2 mM) or high concentration (NaNO_3_ = 12 mM) of nitrate in the culture medium did not significantly affect eGFP accumulation in the culture media. This finding suggests that increasing nitrate availability beyond 1.2 mM did not further enhance P_NR_-driven recombinant protein expression under the tested conditions. In contrast, light intensity (150 µmol photons m^− 2^ s^− 1^ vs. 30 µmol photons m^− 2^ s^− 1^) enhanced eGFP accumulation in the medium, suggesting that PPFD is a key modulator of NR driven transcription and protein production and/or secretion. Importantly, the highest levels of recombinant protein productivity were achieved under culture in Natural Sea Water, indicating that its complex composition may provide micronutrients or cofactors essential for optimal expression and accumulation in the medium. These findings position Natural Sea Water as a valuable reference for the development of optimized artificial media. Future work should aim to gradually refine the composition of synthetic sea water to replicate the growth and productivity benefits observed with Natural Sea Water. While this study was conducted under controlled laboratory conditions, further investigations are required to evaluate additional physicochemical factors, such as salinity, light quality, photoperiod, assess scalability, and elucidate the molecular mechanisms underlying the observed effects through transcriptional analyses. Such studies will improve our understanding of the effects of environmental and nutritional parameters and support the development of efficient and scalable recombinant protein production systems, further establishing *P. tricornutum* as a promising host for industrial biotechnology applications.

## Supplementary Information

Supplementary Material 1.

## Data Availability

The data can be available upon request.
