# Supplementary material for: Enhancing recombinant eGFP secretion in P. tricornutum by optimizing culture conditions
Source: Microb Cell Fact. 2026 Jul 16;25:205. doi: 10.1186/s12934-026-03061-3 (PMC13656395; doi:10.1186/s12934-026-03061-3)
Supplement: Supplementary file 1 [file 12934_2026_3061_MOESM1_ESM.docx]

**Enhancing recombinant eGFP secretion in *P. tricornutum* by optimizing culture conditions**

Nesrine GARGOUCH^1^, Aurélie CHARRIER², Charlotte TOUSTOU^1^, Gaël BOUGARAN², Bruno SAINT-JEAN^3^ and Muriel BARDOR^1,4^

^1^ Université de Rouen Normandie, Laboratoire GlycoMEV UR 4358, SFR Normandie Végétal FED 4277, Innovation Chimie Carnot, F-76000 Rouen, France

² IFREMER, PHYTOX, Laboratoire PHYSALG, F-44000 Nantes, France

^3^ IFREMER, PHYTOX, Laboratoire GENALG, F-44000 Nantes, France

^4^ ALGA BIOLOGICS, 25 Rue Tesnières, 76821 Mont Saint Aignan and 1 rue de l’industrie, 76380 Canteleu, France

*Correspondence to nesrine.gargouch@univ-rouen.fr

**Figure S1**. Map of the NR-eGFPsec expression vector designed for eGFP production under the control of the nitrate reductase (NR) promoter.

The vector contains an ampicillin resistance (eZ_AmpR) for selection in *E. coli*, and a zeocin resistance gene (ZeocinR) for selection of *P. tricornutum* transformants. The eGFP coding sequence (light green) is expressed under the control of the NR promoter (green) and NR terminator (red) (GenBank: JN180663.1)

**Figure S1:** Effect of nitrate concentration on cell-normalized eGFPsec accumulation in the medium by the eGFPsec strain cultivated in Instant Ocean medium.

Cell-normalized extracellular eGFP fluorescence intensity measured in the corresponding cell-free medium of cultures supplemented with 1.2 mM (black histogram) or 12 mM (gray histogram) NaNO_3_.

Fluorescence values were normalized to the corresponding cell density measured at each sampling time.

Histogram were plotted using GraphPad Prism software (version 8.0.2) and error bars indicate standard deviation calculated from three independent biological replicates (n = 3)

**Figure S2:** Effect of irradiance intensity on cell-normalized eGFPsec accumulation in the medium by the eGFPsec strain cultivated in Instant Ocean medium complemented with Conway.
Cell-normalized eGFP fluorescence intensity measured in the corresponding cell-free medium of cultures at different irradiances 30, 90, 150 and 250 µmol photons m^-2^ s^-1^

Fluorescence values were normalized to the corresponding cell density measured at each sampling time.

Histogram were plotted using GraphPad Prism software (version 8.0.2) and error bars indicate standard deviation calculated from three independent technical replicates (n = 3)

**Figure S3**: Evaluation of the influence of culture medium and pH regulation on the accumulation in the medium of eGFP.

Cell-normalized eGFP fluorescence intensity measured in cell-free medium (A) in Salt Instant Ocean medium under unregulated and regulated (pH = 8) pH conditions.

Cell-normalized eGFP fluorescence intensity measured in cell-free medium (B) in natural sea water under unregulated and regulated (pH = 8) pH conditions.

Fluorescence values were normalized to the corresponding cell density measured at each sampling time.

Histogram were plotted using GraphPad Prism software (version 8.0.2) and error bars indicate standard deviation calculated from two independent technical replicates (n = 3)

**Table S1**: ANOVA results showing no significant effect (p > 0.01) of NaNO_3_ enrichment on the growth rate of the eGFP_sec_ strain. Data were obtained from three independent biological replicates (n = 3)

|  | Sum Sq | Df | F Value | Pr(>F) |
| --- | --- | --- | --- | --- |
| Enrichment | 0.091267 | 1 | 1.3568 | 0.3088 |
| Residuals | 0.269067 | 4 |  |  |

Signif. codes: 0 ‘***’ ; 0.001 ‘**’ ; 0.01 ‘*’ ; 0.05 ‘.’ ; 0.1 ‘ ’, 1
